# PB.22. Does mammographic compression force at breast screening influence the likelihood of subsequent screening attendance?

**DOI:** 10.1186/bcr3710

**Published:** 2014-11-03

**Authors:** J Meyer, A Maxwell, E Harkness, S Astley, C Mercer, M Wilson, M Bydder, Y Lim, J Morris

**Affiliations:** 1 University of Manchester Medical School, Manchester, UK; 2 Centre for Imaging Sciences, Institute of Population Health, University of Manchester, UK; 3 Nightingale Centre and Genesis Prevention Centre, University Hospital of South Manchester, Manchester, UK; 4 University Hospital of South Manchester, Manchester, UK

## Introduction

Previous work has demonstrated that approximately 14% of women who attend their first breast screening appointment fail to attend the next appointment. It is well recognised that some women experience marked discomfort or pain during mammography which may be related to the breast compression force applied. This study investigates whether applied compression force is related to the likelihood of attendance for the next scheduled screening mammogram.

## Methods

A search on the national breast screening database identified women aged 46 to 53 who attended for prevalent screening in 2009/10 and their attendance status at the next invited screen was recorded. A total of238 subsequent nonattenders were identified together with a sample of 240 women who had subsequently attended that had a similar age distribution. The compression force used for each of the prevalent screening images for these 478 women was recorded.

## Results

The median age of the women in both groups was 50 years. The mean compression force applied for all images during the prevalent screen was 102.1 N for subsequent attenders and 104.4 N for subsequent nonattenders (*P *= 0.263). There were similarly no significant differences between the two groups for maximum compression force (*P *= 0.410) or compression force for individual views (LMLO, *P *= 0.200; RMLO, *P *= 0.605; LCC, *P *= 0.903; RCC, *P *= 0.246).

## Conclusion

No statistically significant relationship has been established between mammographic compression force and the likelihood of subsequent screening attendance. Other factors such as compression pressure (compression force per unit area of contact) may be more important, and further research is planned to investigate this.

